# Equal Pro-inflammatory Profiles of CCLs, CXCLs, and Matrix Metalloproteinases in the Extracellular Microenvironment *In Vivo* in Human Dense Breast Tissue and Breast Cancer

**DOI:** 10.3389/fimmu.2017.01994

**Published:** 2018-01-16

**Authors:** Annelie Abrahamsson, Anna Rzepecka, Charlotta Dabrosin

**Affiliations:** ^1^Department of Oncology, Linköping University, Linköping, Sweden; ^2^Department of Clinical and Experimental Medicine, Linköping University, Linköping, Sweden; ^3^Department of Radiology, Linköping University, Linköping, Sweden; ^4^Department of Medical and Health Sciences, Linköping University, Linköping, Sweden

**Keywords:** cytokines, mammary gland, microdialysis, chemokines, microenvironment

## Abstract

The inflammatory microenvironment affects breast cancer progression. Proteins that govern the inflammatory response are secreted into the extracellular space, but this compartment still needs to be characterized in human breast tissues *in vivo*. Dense breast tissue is a major risk factor for breast cancer by yet unknown mechanisms and no non-toxic prevention for these patients exists. Here, we used the minimal invasive technique of microdialysis for sampling of extracellular proteins in live tissues *in situ* in breast cancers of women before surgery and in healthy women having dense or non-dense breast tissue on mammography. Proteins were profiled using a proximity extension assay. Out of the 32 proteins assessed, 26 exhibited similar profiles in breast cancers and dense breast tissues; CCL-4, -7, -8, -11, -15, -16, -22, -23, and -25, CXCL-5, -8, -9, -16 as well as sIL-6R, IL-18, vascular endothelial growth factor, TGF-α, fibroblast growth factor 19, matrix metalloproteinase (MMP)-1, -2, -3, and urokinase-type plasminogen activator were all increased, whereas CCL-3, CX3CL1, hepatocyte growth factor, and MMP-9 were unaltered in the two tissues. CCL-19 and -24, CXCL-1 and -10, and IL-6 were increased in dense breast tissue only, whereas IL-18BP was increased in breast cancer only. Our results provide novel insights in the inflammatory microenvironment in human breast cancer *in situ* and define potential novel therapeutic targets. Additionally, we show previously unrecognized similarities of the pro-inflammatory microenvironment in dense breast tissue and breast cancer *in vivo* suggesting that anti-inflammatory breast cancer prevention trials for women with dense breast tissue may be feasible.

## Introduction

Inflammation is one of the hallmarks of cancer initiation and progression (1). The importance of inflammation in tumor growth and metastasis has been described in numerous cancer types, including breast cancer (2, 3). Inflammatory bioactive molecules such as interleukins and tumor necrosis factor, released into the interstitial fluid can be tumor promoting by affecting angiogenesis, cancer cell proliferation, invasiveness, and metastasis (4–6). Additionally, inflammation, which also is related to oxidative stress, might foster the development of incipient *in situ* cancers into invasive clinically important cancers (7).

In breast cancer, some epidemiological studies support this concept whereas others have failed to show a correlation between regular use of non-steroidal anti-inflammatory drugs (NSAIDs) and progression of this disease (8–12). The malignant potential of atypical breast epithelial cells is dependent on the local microenvironment but this compartment still needs to be characterized in human breast cancer and normal breast tissue with increased risk of cancer.

Increased mammographic density is a major independent risk factor for breast cancer (13). A fourfold increased risk of developing breast cancer has been shown for women with dense breast tissue, and absolute non-dense area seems to be independently and inversely associated with breast cancer risk (13, 14). The biological differences between the two types of breast densities and how it might contribute to breast cancer risk are, however, poorly understood. Dense breast tissue contains higher amounts of stroma, including collagen, and less fat but the proportion of epithelial cells, which is only 1–6% of the tissue, seems to be unrelated to breast density (13, 15–17). A few studies have shown that dense breast tissue may be associated with increased inflammation and an altered metabolic profile (18–20).

Although mammography screening programs and improved treatments have reduced the death rate of breast cancer, prevention strategies are still needed for this disease that affects more than 10% of all women (21). In some countries, the anti-estrogen tamoxifen is approved for prevention, but this therapy is associated with severe side-effects, such as thromboembolism, endometrial cancer, and low quality of life (21, 22). Thus, providing effective and non-toxic breast cancer prevention for women at increased risk is clearly needed. Additionally, tamoxifen treatment to women with breast cancer will only reduce the risk of recurrence by 30–50% (23). Hence, increased knowledge of the biology of breast cancer and dense breast tissue is key for finding novel therapeutic targets.

The tissue microenvironment, including the interstitial fluid, contains essential components that are important for the facilitation of cancer growth and is a key determinant for cancer progression. The interstitial fluid represents approximately one-third of the total body fluid but to date this compartment still needs to be characterized. The homeostasis of the microenvironment is regulated by soluble factors in the interstitial fluid and one major difficulty in studying this, is the collection of components from this compartment. Therefore, we used microdialysis as an *in vivo* approach for sampling of extracellular molecules from the interstitial fluid directly in live tissue *in situ*. Breast cancer patients and healthy women with either dense or non-dense breast tissue were included in the study. The two main objectives of the study were to characterize the inflammatory microenvironment in human breast cancers and to investigate any resemblance of the microenvironment in dense breast tissue and breast cancer. We show that out of the 32 assessed proteins related to inflammation, 26 exhibited similar regulation in dense breast tissue as in breast cancer. Our results provide novel biological understanding of human breast cancer defining several targets for further immunotherapy research and indicate that investigations of targeting inflammation as a prevention strategy in women with dense breast tissue may be feasible.

## Materials and Methods

### Study Populations

The study was carried out in accordance with the Declaration of Helsinki and the Regional Ethical Review Board of Linköping, Sweden, approved the study. All subjects gave written informed consent. A total of 51 women were included in two different patient cohorts. The first cohort consisted of 12 women who had breast cancer and were investigated before surgery. Tumor histology, size, immunohistochemistry for estrogen receptor (ER) and progesterone receptor (PR), and HER-2 receptor, and Nottingham histological grade according to the Elston Ellis scoring system were determined at the Department of Pathology and Cytology, University Hospital of Linköping, Table 1. For the second cohort, 39 healthy postmenopausal women (55 years of age or older) were consecutively recruited from the screening mammography program at Linköping University Hospital as previously described (20). The regular mammograms of the women were assessed by one experienced observer (Anna Rzepecka) according to the Breast Imaging Reporting and Data System (BI-RADS) density scale (24), and breast densities were categorized as either BI-RADS A (entirely fatty non-dense breasts) or BI-RADS D (extremely dense). An MRI was performed on these women to confirm the initial breast density assessment, as described previously (20). None of the healthy women had a history of previous breast cancer or benign breast disease. In addition, none of the 51 investigated women were currently using (or had used within the previous 3 months) hormone replacement therapy, anti-estrogen therapies, including selective ER modulators or degraders, or regularly used of NSAIDs, including over the counter preparations.

**Table 1:** Characteristics of patients subjected to intratumoral microdialysis.

Patient	Age	Tumor size	Grade (NHG)	ER (%)	PR (%)
1	70	22	2	>50	>50
2	68	24	2	>50	>50
3	52	25	3	>50	10–50
4	78	28	2	>50	>50
5	62	21	2	>50	>50
6	63	19	2	>50	>50
7	45	40	3	0	0
8	61	25	2	>50	>50
9	48	30	2	>50	>50
10	73	30	2	>50	<5
11	57	27	1	>50	>50
12	66	60	2	>50	>50

*ER, estrogen receptor; PR, progesterone receptor; NHG, Nottingham histological grade*.

*All cancers were HER-2 negative*.

### Microdialysis Procedure

The women with ongoing breast cancer were investigated before surgery; one microdialysis catheter was inserted within the cancer tumor and the other microdialysis catheter was inserted into normal adjacent breast tissue. In the healthy volunteer women investigated in normal breast tissue, the microdialysis catheter was placed in the upper lateral quadrant of the left breast directed toward the nipple as previously described (25–34).

Prior insertion of the microdialysis catheters, 0.5 ml lidocain (10 mg*/*mL) was administrated intracutaneously. Microdialysis catheters (71*/*M Dialysis AB, Stockholm, Sweden), which consists of a tubular dialysis membrane (diameter 0.52 mm, 100,000 atomic mass cutoff) glued to the end of a double-lumen tube (80 mm long × 0.8 mm in diameter), were inserted *via* a splitable introducer (M Dialysis AB), connected to a microinfusion pump (M Dialysis AB) and perfused with NaCl 154 mmol*/*L and hydroxyethyl starch 60 g/L (Voluven^®^, Fresenius Kabi, Uppsala, Sweden), at a perfusion rate of 0.5 µL/min. 20 and 10 mm membranes were used in the cohort of various breast densities and breast cancer patients, respectively. After a 60-min equilibration period, the outgoing perfusate was stored at −80°C for subsequent analysis.

### Protein Quantifications

The samples were analyzed by using a multiplex proximity extension assay (Olink Bioscience, Uppsala Sweden). In brief, 1 µL sample was incubated in the presence of proximity antibody pairs tagged with DNA reporter molecules. Once the pair of antibodies is bound to their corresponding antigens, the respective DNA tails form an amplicon by proximity extension, which was quantified by high-throughput real-time PCR (BioMark™ HD System, Fluidigm Corporation). The generated fluorescent signal directly correlates with protein abundance. The output from the Proseek Multiplex protocol is in quantitation cycles produced by the BioMark’s Real-Time PCR Software. To minimize variation within and between runs, the data are normalized using both an internal control (extension control) and an interplate control, and then transformed using a pre-determined correction factor. The pre-processed data were provided in the arbitrary unit normalized protein expression (NPX) on a log_2_ scale, which were then linearized by using the formula 2^NPX^. A high NPX value corresponds to a high protein concentration. However, the value is a relative quantification meaning that no comparison of absolute levels between different proteins can be made.

### Statistical Analyses

Statistical analyses were performed using paired Wilcoxon matched-pairs signed rank test and unpaired Mann–Whitney *U* test. A *p* < 0.05 was considered as statistically significant. Statistics were performed with Prism 7.0 (GraphPad software).

## Results

As shown in Table 1, 11 out of the 12 breast cancers were ER positive. None of the cancers overexpressed HER-2. As previously reported there were no significant differences in age, years since menopause, BMI, or plasma estradiol levels between the healthy women with dense vs. non-dense breast tissues (20). There were no subsequent complications after the microdialysis investigations in either patient group.

### CCLs in Dense Breast Tissue and Breast Cancers

**Figure 1:**
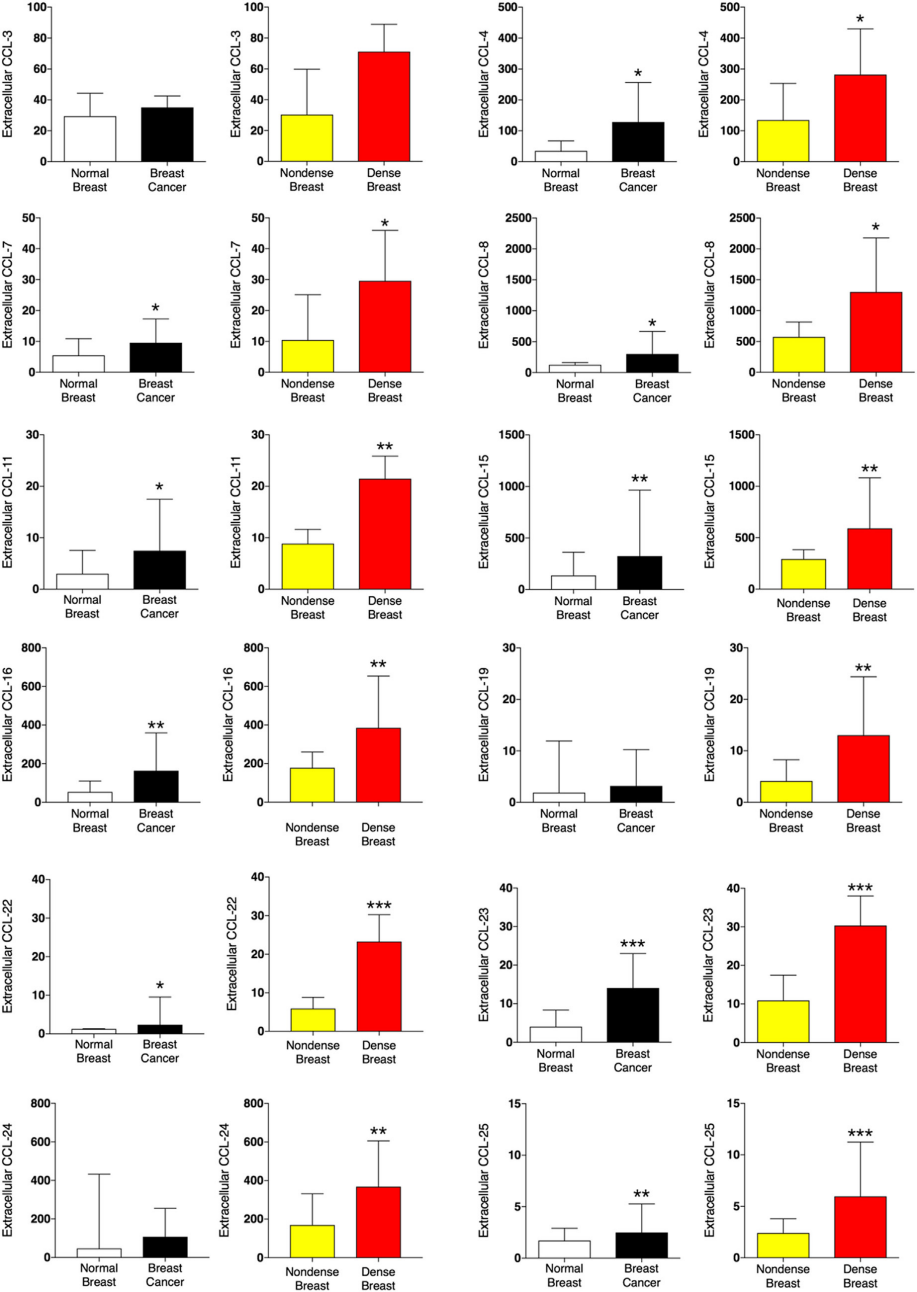
Extracellular levels of CCLs *in vivo* in breast cancers and healthy normal breast tissue with different densities. 51 women were investigated using microdialysis; 12 breast cancer patients underwent microdialysis before surgery. One catheter was inserted into the breast cancer (black bars) and another into adjacent normal breast tissue (white bars). 39 postmenopausal healthy volunteer women, attending the regular mammography-screening program and were categorized as either having dense or non-dense breasts underwent microdialysis of their left breast. Women with dense breasts (*n* = 20) are depicted in red and women categorized as non-dense (*n* = 19) are depicted in yellow. Data represent protein abundance in linear values (2^NPX^ as described in the Section “Materials and Methods”). Graphed data are presented as median with 95% CI (*P < 0.05, **P < 0.01, ***P < 0.001).

A panel of 12 CCLs was analyzed (Figure 1). Ten out of the 12 CCLs were regulated in a similar fashion in dense breast tissue and breast cancer. In breast cancer CCL-4, -7, -8, -11, -15, -16, -22, -23, and -25 exhibited increased levels compared with normal adjacent breast tissue, whereas no significant differences were found of CCL-3, -19, and -24. In dense breast tissue, CCL-4, -7, -8, -11, -15, -16, -19, -22, -23, -24, and CCL-25 were increased compared with non-dense breast tissue. No significant differences were found in the levels of CCL-3 within the breast cancer cohort, or the healthy women cohort. Two CCLs were differently regulated; CCL-19 and -24 were unaltered in breast cancers but increased in dense breast tissue.

### CXCLs and CX3CL1 in Dense Breast Tissue and Breast Cancers

**Figure 2:**
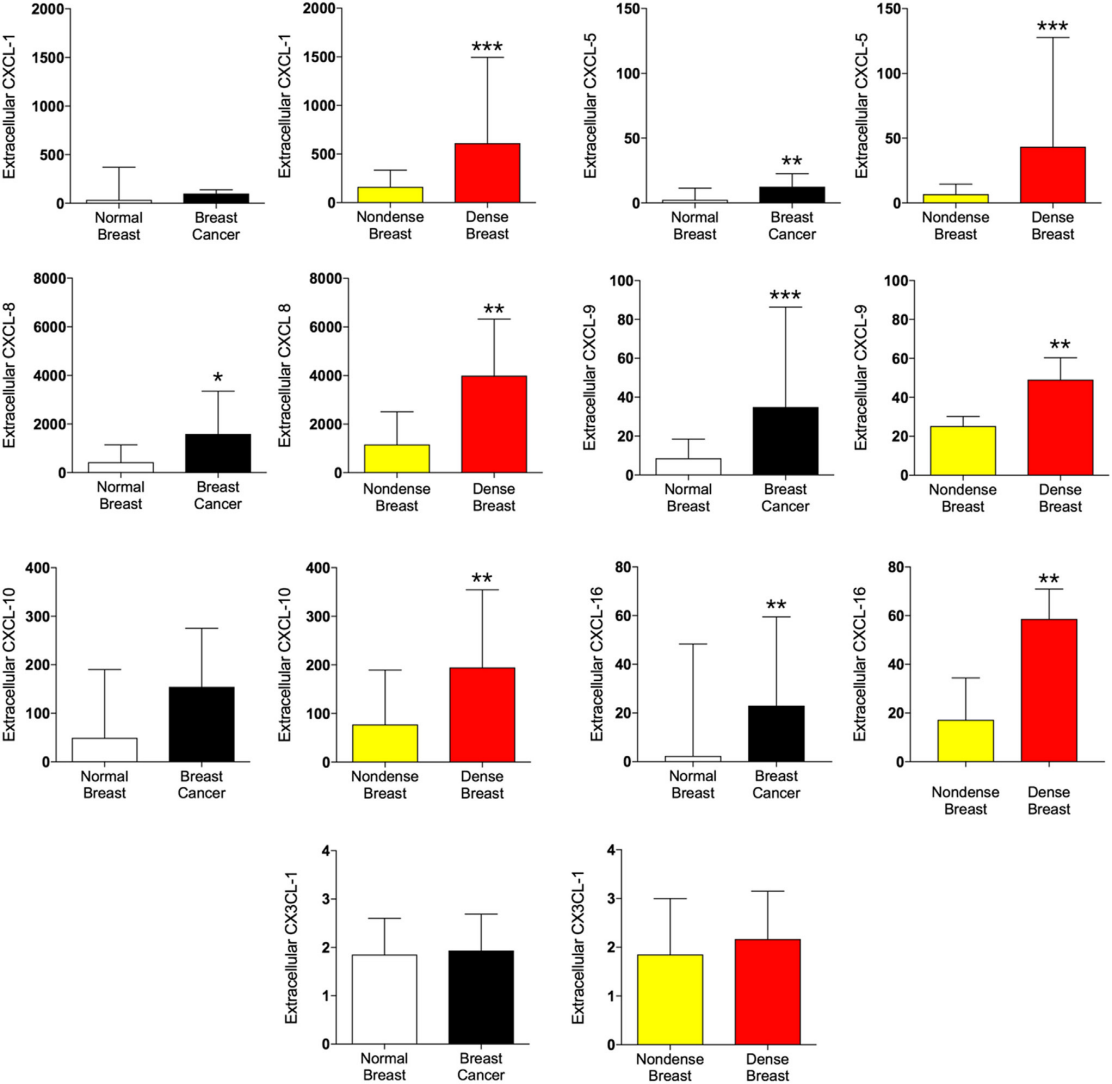
Extracellular levels of CXCL, CX3CL1, and IL-18 *in vivo* in breast cancers and healthy normal breast tissue with different densities. 51 women were investigated using microdialysis; 12 breast cancer patients underwent microdialysis before surgery. One catheter was inserted into the breast cancer (black bars) and another into adjacent normal breast tissue (white bars). 39 postmenopausal healthy volunteer women, attending the regular mammography-screening program and were categorized as either having dense or non-dense breasts underwent microdialysis of their left breast. Women with dense breasts (*n* = 20) are depicted in red and women categorized as non-dense (*n* = 19) are depicted in yellow. Data represent protein abundance in linear values (2^NPX^ as described in the Section “Materials and Methods”). Graphed data are presented as median with 95% CI (*P < 0.05, **P < 0.01, ***P < 0.001).

As shown in Figure 2, CXCL-5, -8, -9, and -16 were increased in breast cancer and dense breast tissue compared with the corresponding normal tissues. Interestingly, CXCL-1 and CXCL-10 were unaltered in breast cancers whereas significant increased levels were detected in dense breast tissue. No change of CX3CL1 was found in either tissue.

### IL-6, IL-18, and Pro-Tumorigenic Proteins in Dense Breast Tissue and Breast Cancers

IL-6 was unaltered in breast cancer but significantly increased in dense breast tissue, whereas IL-6RA was increased in both tissues. Increased levels of IL-18 were found in dense breast tissue as well as in breast cancer, but IL-18BP was increased in breast cancer only.

**Figure 3:**
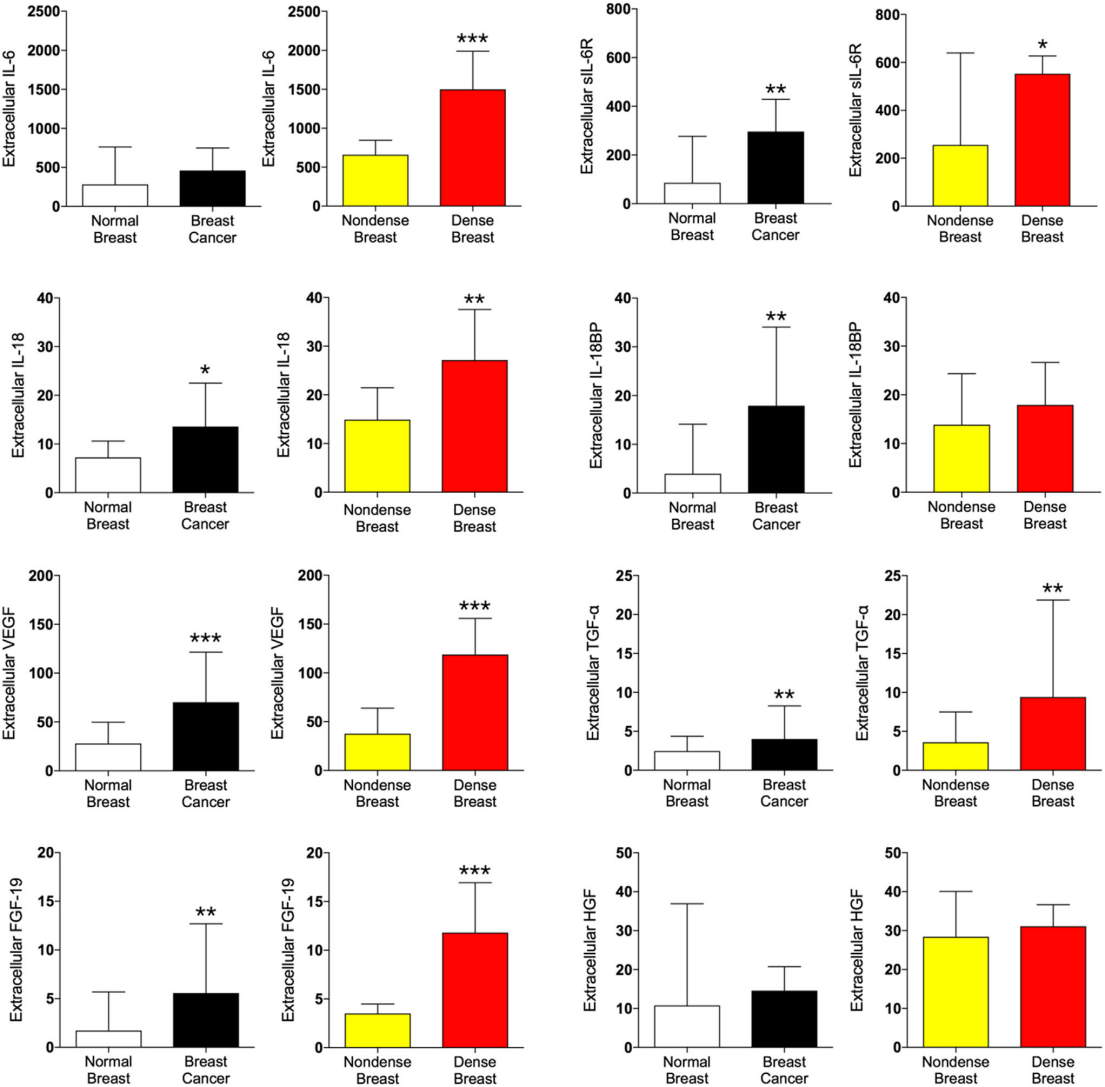
Extracellular levels of pro-tumorigenic proteins *in vivo* in breast cancers and healthy normal breast tissue with different densities. 51 women were investigated using microdialysis; 12 breast cancer patients underwent microdialysis before surgery. One catheter was inserted into the breast cancer (black bars) and another into adjacent normal breast tissue (white bars). 39 postmenopausal healthy volunteer women, attending the regular mammography-screening program and were categorized as either having dense or non-dense breasts underwent microdialysis of their left breast. Women with dense breasts (*n* = 20) are depicted in red and women categorized as non-dense (*n* = 19) are depicted in yellow. Data represent protein abundance in linear values (2^NPX^ as described in the Section “Materials and Methods”). Graphed data are presented as median with 95% CI (*P < 0.05, **P < 0.01, ***P < 0.001).

In addition to inflammatory cytokines, proteins associated with angiogenesis and cancer progression were also analyzed. As shown in Figure 3, vascular endothelial growth factor (VEGF), transforming growth factor-α (TGF-α), and fibroblast growth factor 19 (FGF-19) all demonstrated increased levels both in dense breast tissue and in breast cancer whereas no difference of hepatocyte growth factor (HGF) levels were found in either tissue.

### Proteases in Dense Breast Tissue and Breast Cancers

**Figure 4:**
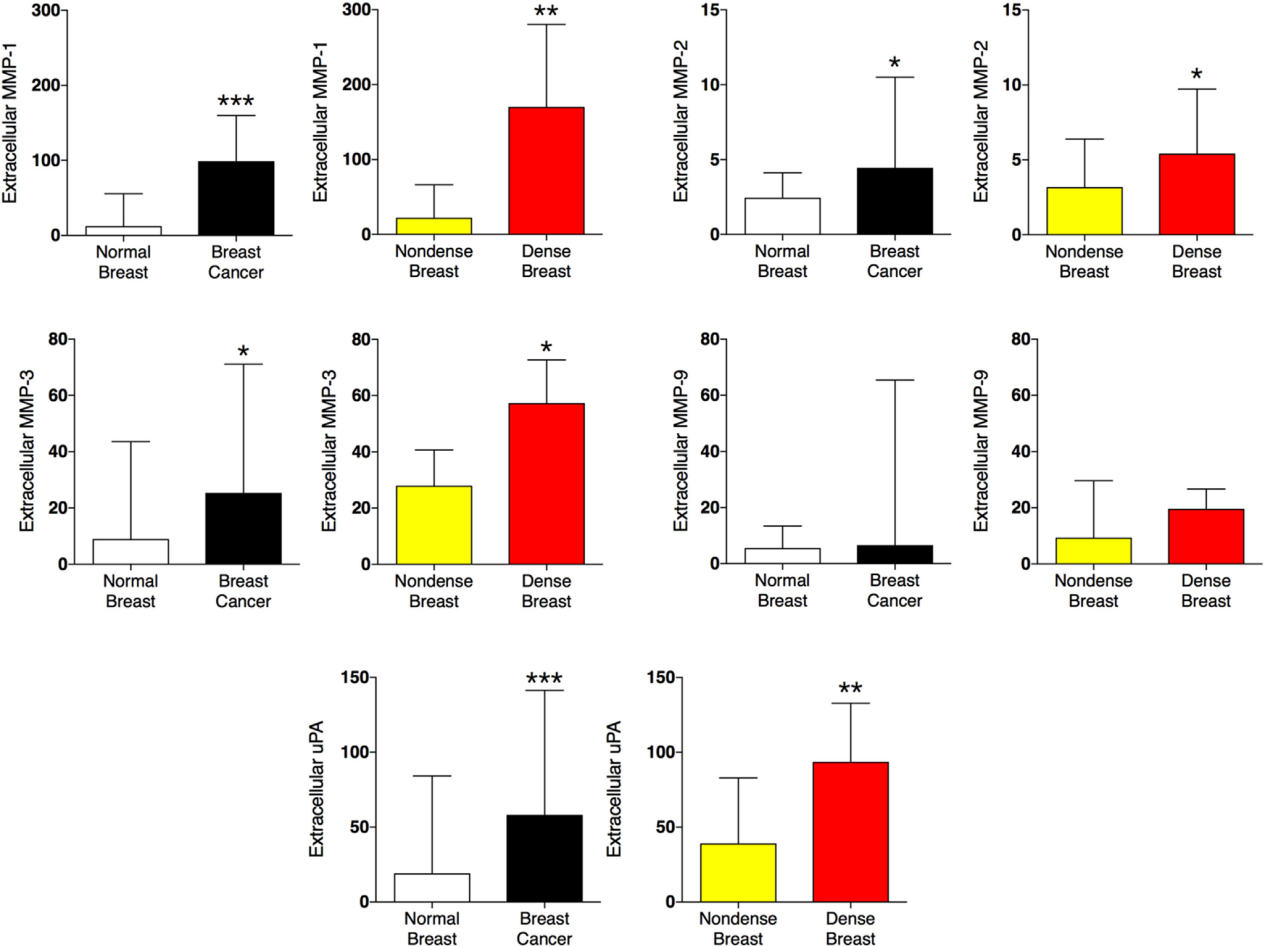
Extracellular levels of proteases *in vivo* in breast cancers and healthy normal breast tissue with different densities. 51 women were investigated using microdialysis; 12 breast cancer patients underwent microdialysis before surgery. One catheter was inserted into the breast cancer (black bars) and another into adjacent normal breast tissue (white bars). 39 postmenopausal healthy volunteer women, attending the regular mammography-screening program and were categorized as either having dense or non-dense breasts underwent microdialysis of their left breast. Women with dense breasts (*n* = 20) are depicted in red and women categorized as non-dense (*n* = 19) are depicted in yellow. Data represent protein abundance in linear values (2^NPX^ as described in the Section “Materials and Methods”). Graphed data are presented as median with 95% CI (*P < 0.05, **P < 0.01, ***P < 0.001).

Proteolysis in the tissue microenvironment may influence the release of local inflammatory mediators in the tissue microenvironment. In Figure 4, the five proteases detected in the present study were altered in a similar fashion in both tissues; matrix metalloproteinase (MMP)-1, 2, 3, and urokinase-type plasminogen activator (uPA) were increased whereas MMP-9 was unaltered.

## Discussion

Here, we present novel data of the local inflammatory microenvironment in human breast cancer *in vivo*. Additionally, to the best of our knowledge, this is the first report showing the similarity of the *in vivo* inflammatory cytokine profile in dense breast tissue and breast cancer. Out of 32 pro-inflammatory proteins analyzed, 26 exhibited similar profiles of the levels in dense breast tissue vs. non-dense breast tissue, and breast cancer tissue vs. normal adjacent breast tissue.

A major strength of our study is that we sampled the proteins directly from tissues in patients and healthy volunteers *in situ*. Only by using a minimally invasive technique such as microdialysis, samples from healthy breast tissue in women not scheduled for any clinically advised procedure can be obtained. *In vivo* studies of the tissue microenvironment, where cell–cell interactions are communicated by soluble factors released into the extracellular compartment, are especially important. Commonly used laboratory techniques such as mRNA quantification and immunohistochemistry are based on whole tissue analyses and, therefore, detect cellular events rather than soluble extracellular molecules. Unlike *ex vivo* sampling of the tumor interstitial fluid, for which surgically removed tissue is cut into pieces before incubation in a solution to retrieve the remaining proteins, microdialysis allows sampling of the extracellular molecules directly from live tissue without any manipulation. The draw-back of such studies is that a limited number of subjects can be included in the time-consuming *in vivo* sampling with the complicated logistics.

It has previously been shown that several of the CC chemokines play an important role for breast cancer progression and immune cells recruited by these chemokines are implicated in enhancing cancer growth and resistance to therapy (35–40). Interestingly, CCL-3, which was unaltered in both breast cancer and dense breast tissue, has been shown to be important for breast cancer progression and metastases in experimental animal models (38, 40). Our human data do not support an extracellular upregulation of this chemokine in breast cancers *in vivo*.

Chemokines can play dual roles in tumor development. The chemokines CXCL-9 and CXCL-10 are involved in the activation of antitumor Th1 cells, but they also function as potent angiostatic factors (41, 42). On the other hand, the neutrophil chemotactic chemokines, CXCL-1 and CXCL-8, have been shown to be pro-tumorigenic and pro-angiogenic by their ability to recruit pro-tumorigenic neutrophils into cancer tissue (43–47). CXCL-9 was increased in both breast cancer and in dense breast tissue suggesting that among an upregulation of pro-inflammatory cytokines other may balance the net results of the inflammatory response of the tissue. Surprisingly, no significant upregulation of the extracellular CXCL-1 was detected in breast cancers whereas dense breast tissue exhibited at least three times higher levels than non-dense breast tissue. In several studies CXCL-8 has been shown to be a key player in the inflammatory/angiogenic microenvironment including increasing invasion and chemotaxis of breast cancer stem cells (44, 45). CXCL-8 also regulates the release of VEGF, which is one of the most potent and specific angiogenic factors. Several isoforms of VEGF exist but the main bioactive forms are freely diffusible proteins in the extracellular space and have greater angiogenic and tumorigenic properties than the heparin-bound isoforms (48). We have previously shown that direct measurements of soluble VEGF locally in tissues accurately reflect the amount of bioactive protein released *in situ* (30, 49, 50). As expected, and in line with previous data, increased VEGF levels were found in both breast cancers and dense breast tissue compared with their normal tissue counterparts.

The pleiotropic cytokine IL-6 has been implicated in several cancer forms, including breast cancer. Circulating levels of IL-6 has been associated the progression of breast cancer and with advanced disease (51). In our data set, no increased levels of IL-6 were detected in breast cancer, whereas in dense breast tissue the IL-6 levels were increased. However, significantly increased levels of extracellular sIL-6R were detected in both tissues. In a classic signaling, IL-6 binds to the IL-6 receptor, and this complex bind to glycoprotein 130 receptors in the cell membrane for the initiation of downstream signaling. However, a trans-signaling may also occur when an extracellular IL-6/sIL-6R complex is formed which then binds to membrane glycoprotein 130 and generate a downstream signaling (52). The IL-6 membrane receptor is expressed in hepatocytes and immune cells only, thus, the classic signaling is limited to these cells. As the membrane glycoprotein 130 is expressed in all cells types a trans-signaling by sIL-6R may be elicited universally and may, therefore, have a higher impact of the immune response and thus play a major role in the IL-6 signaling.

The pro-inflammatory cytokine IL-18 has been associated with both pro- and antitumorigenic properties (53). IL-18BP, a natural inhibitor of IL-18, has high affinity for mature IL-18 and blocks its interaction with the IL-18 receptor (53). Our results revealed increased levels of both IL-18 and IL-18BP in breast cancer whereas in dense breast tissue IL-18 alone was increased. Whether this is an advantage or disadvantage regarding breast cancer progression remains to be elucidated when the exact biologic role of IL-18 in cancer development is determined.

We also measured three key pro-tumorigenic growth and angiogenic factors associated with inflammation, TGF-α, FGF-19, and HGF, and showed that both TGF-α and FGF-19 exhibited increased levels in both breast cancer and dense breast tissue compared with their normal tissue counterparts. TGF-α is a natural ligand for the EGFR, which is overexpressed in many tumors and plays a central role in cancer development by promoting cell proliferation and angiogenesis (54). FGFs have been demonstrated to increase cancer cell survival in experimental murine breast cancer models (55). Our present data revealed that the FGF-19 levels were twice as high in breast cancer and tripled in dense breast tissue, compared with the normal tissues within each cohort, suggesting that FGF-19 may be a clinically relevant protein to target also in human breast tissue.

Hepatocyte growth factor has been implicated in the progression of experimental breast cancer (56). Our data did not reveal any differences in HGF levels. The association of HGF and breast cancer progression has primarily been shown in triple-negative breast cancers; our data are mainly based on ER+ breast cancer, whether HGF is increased in ER− human breast cancer remains to be elucidated.

Proteolysis is a regulatory pathway that influences the local tissue inflammation by degrading and remodeling of the microenvironment, and by cleavage of precursors of cytokines and growth factors. Additionally, several cytokines may induce cells in the microenvironment to secrete MMPs (57, 58). Upregulation of MMPs and paradoxically TIMPs has been correlated with tumor aggressiveness of various cancer forms, including breast cancer (59–64). However, the biological function of MMPS is complex as it has been demonstrated that individual MMPs and TIMPs may either promote or inhibit tumor progression and MMP inhibition has also failed as antitumor therapy in clinical trials (65–68). Here, we show that MMP-1 was increased in both breast cancer and dense breast tissue. MMP-1 has been significantly correlated with the development of brain metastasis in breast cancer patients and with a release of CCL-7 (69). In addition, MMP-1 has been associated with multi-drug resistance in experimental breast cancer (70). Its role in clinical breast cancer patients remains to be determined but our data suggest that indeed, MMP-1 is increased in human breast cancer. MMP-2 and MMP-3 were also increased in both tissues whereas the levels of MMP-9 were unaltered. Regarding MMP-9, we and others, have shown that increased MMP-9 activity leads to tumor regression (67, 68, 71–75). The data in the present study suggest that MMP-9 may not be a major contributor to breast cancer progression/inhibition in human breast tissue *in vivo*.

Urokinase-type plasminogen activator is a serine protease that has been shown to be involved in multiple steps of breast cancer progression and it has also been independently associated with adverse outcome in breast cancer patients (76). As expected, uPA was significantly increased in breast cancer but in addition, we made the novel findings that uPA was also increased in dense breast tissue.

In conclusion, our data have revealed that the microenvironment in dense breast tissue exhibits similar profiles of inflammatory proteins as breast cancer [>80% of the measured proteins (26/32)]. Whether a tissue microenvironment exerts a pro- or anti-inflammatory response depends on the balance between inflammatory proteins, and our data suggest that the scale of inflammatory biomarkers is tipped into a pro-inflammatory microenvironment in both dense breast tissue and in breast cancer. It may, therefore, be hypothesized that if atypical epithelial cells arise in dense breast tissue a pro-inflammatory extracellular microenvironment would be more permissive for a continuous expansion of these cells into a clinically important breast cancer. Given the toxicity of the only approved prevention drug tamoxifen, other novel preventive approaches for breast cancer are needed. Our study provides mechanistic support for prevention strategies targeting inflammation in women with dense breast tissue. Thus, studies investigating whether the local tissue microenvironment in dense breast tissue can be modulated with anti-inflammatory therapy are warranted. In addition, our results provide novel biological understanding of human breast cancer defining several targets for further cancer immunotherapy research.

## Ethics Statement

The study was carried out in accordance with the Declaration of Helsinki and the Regional Ethical Review Board of Linköping, Sweden, approved the study. All subjects gave written informed consent.

## Author Contributions

All authors collaborated on the study conception, study design, and data interpretation. AR assessed mammographic densities. CD performed all microdialysis experiments. AA carried out the sample preparation. AA and CD performed data analysis and drafted the manuscript. All authors read and approved the final version of the manuscript.

## Conflict of Interest Statement

None of the authors have any financial, commercial, or other conflicts of interest to disclose.
